# Severe bilateral papilledema after sigmoid sinus constriction surgery: a case report

**DOI:** 10.1186/s12886-023-03252-1

**Published:** 2023-12-08

**Authors:** Xi Chen, Yizhou Ren, Fang Chen

**Affiliations:** 1 Department of Ophthalmology, Northern Jiangsu People’s Hospital, Yangzhou, Jiangsu Province China; 2 Clinical Medical College, Yangzhou University, Yangzhou, Jiangsu Province China

**Keywords:** Papilledema, Intracranial Hypertension, Pulsating tinnitus, Sigmoid sinus constriction Surgery, Sigmoid sinus stenosis, Case report

## Abstract

**Background:**

Papilledema is a common sign of various diseases in the eye. It could result from any conditions of increased intracranial pressure (ICP). Underlying the etiology of papilledema and appropriate treatment in time is essential.

**Case report:**

We present a case of severe bilateral papilledema after sigmoid sinus constriction surgery. A 25-year-old female presented with a 1-month history of bilateral blurred vision, headache, and vomiting. The patient had a history of right-side sigmoid sinus constriction surgery for pulsatile tinnitus (PT) one month before in another hospital. Fundus examination showed severe bilateral papilledema. Lumbar puncture showed an elevated cerebrospinal fluid (CSF) opening pressure of 29 cm H_2_O. Neuroimaging examination demonstrated the right sigmoid sinus filling defect as changes after surgery. We referred the patient to the initial surgeon, who repaired the sigmoid sinus on the right side by removing the implanted gelatin sponge, as diuretic treatment could not be effective. Intracranial hypertension symptoms and signs improved soon after eliminating sigmoid sinus stenosis. Neuroimaging showed resolved right sigmoid sinus stenosis after the second surgery. CSF opening pressure was 14.5 cm H_2_O at the 1-month follow-up. Fundus examination showed entirely resolved papilledema. Three years of follow-up showed no recurrence.

**Conclusions:**

This is the first clinical report of intracranial hypertension associated with sigmoid sinus constriction surgery. Although rare, rapid detection and adequate etiology management could lead to a good prognosis. It highlights the need for ophthalmologists to be aware of the diagnostic approach to papilledema and enhance cooperation with multidisciplinary departments. The most likely cause of the intracranial hypertension was dominant sinus surgical constriction by mechanical external compression, as confirmed by the complete clinical remission following the second operation to remove the implanted gelatin sponge. Thus, this case also highlights the importance of selecting the appropriate therapeutic option for PT. Surgical sinus constriction should no longer be considered a viable option for PT treatment.

## Background

Papilledema is optic nerve head edema secondary to elevated intracranial pressure (ICP) [1]. Idiopathic intracranial hypertension (IIH) is a neurologic syndrome characterized by increased ICP without an identifiable cause. The clinical manifestations of secondary intracranial hypertension (SIH) and IIH are similar. Ocular symptoms usually include decreased central visual acuity, visual field defect, transient visual obscurations, and diplopia [2]. Other symptoms include bilateral or unilateral pulsating tinnitus (PT), gait instability, headache, nausea, vomiting, spontaneous rhinoliquorrhea, dizziness, and disturbed concentration. The diagnosis of IIH is based on the exclusion of SIH [3]. The diagnostic and therapeutic principles for patients with papilledema are to find any underlying cause, protect and save vision, and provide onward care from multidisciplinary, experienced clinicians [3]. Various causes of SIH have been reported, for example, drug-induced SIH (e.g., tetracycline, doxycycline, minocycline), infectious factors (e.g., cryptococcal meningitis, Lyme disease), hormone-related conditions (e.g., primary or secondary aldosteronism), and other diseases (e.g., anemia, rheumatic system diseases) [1, 3–6]. However, no intracranial hypertension was reported related to sigmoid sinus constriction surgery.

Here, we report an uncommon case of severe bilateral papilledema following sigmoid sinus constriction surgery in a 25-year-old female.

## Case presentation

A 25-year-old woman presented to the ophthalmology department complaining of bilateral blurred vision, headache, and vomiting from one month ago. The patient had a history of right-side PT for one year and surgery for PT one month before. Her review of systems was negative. Her family and social history were insignificant. Her body mass index (BMI) was 21 kg/m^2^.

**Fig. 1:**
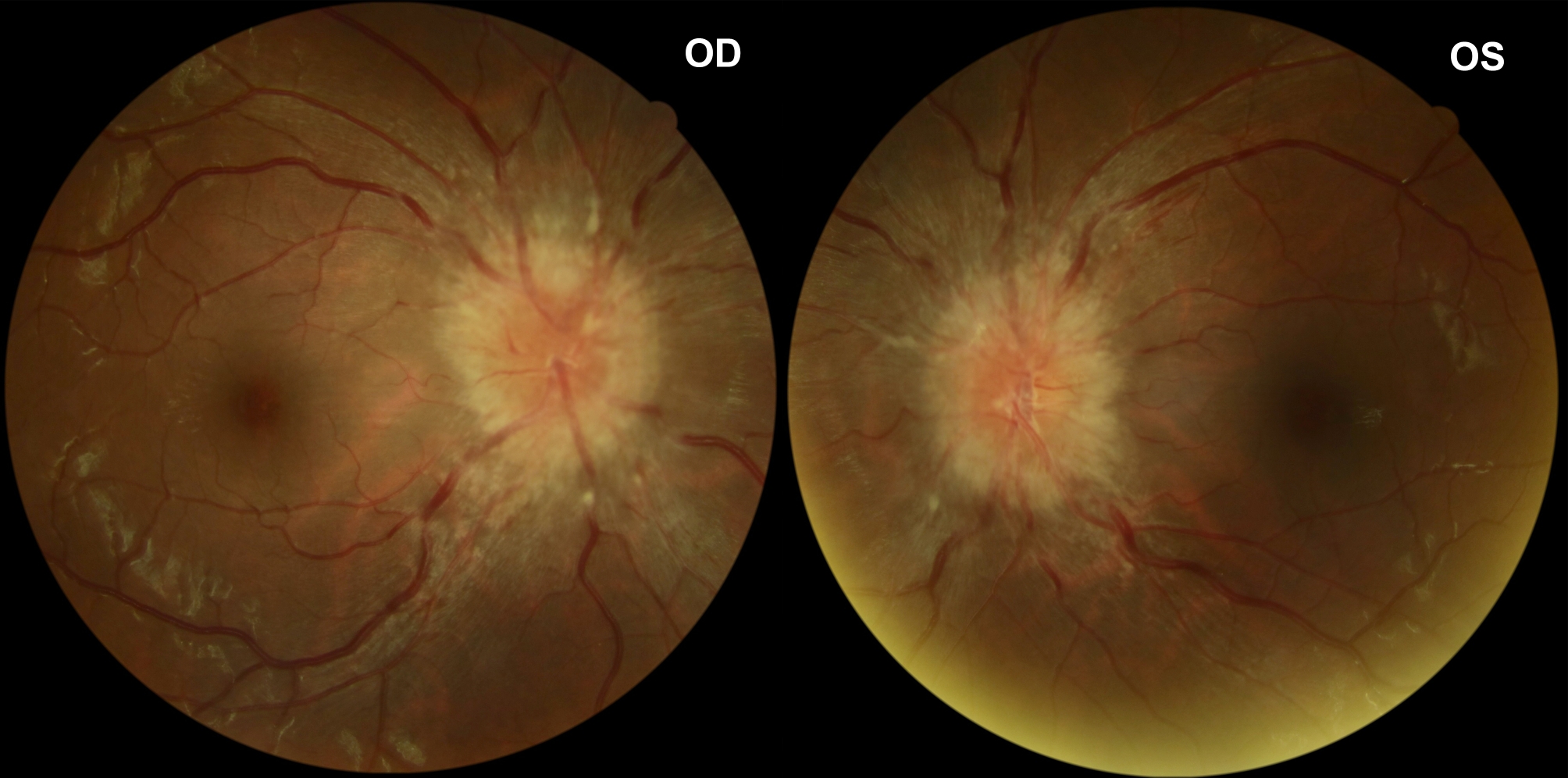
Color fundus photograph: funduscopic photographs of both eyes showing marked papilledema

**Fig. 2:**
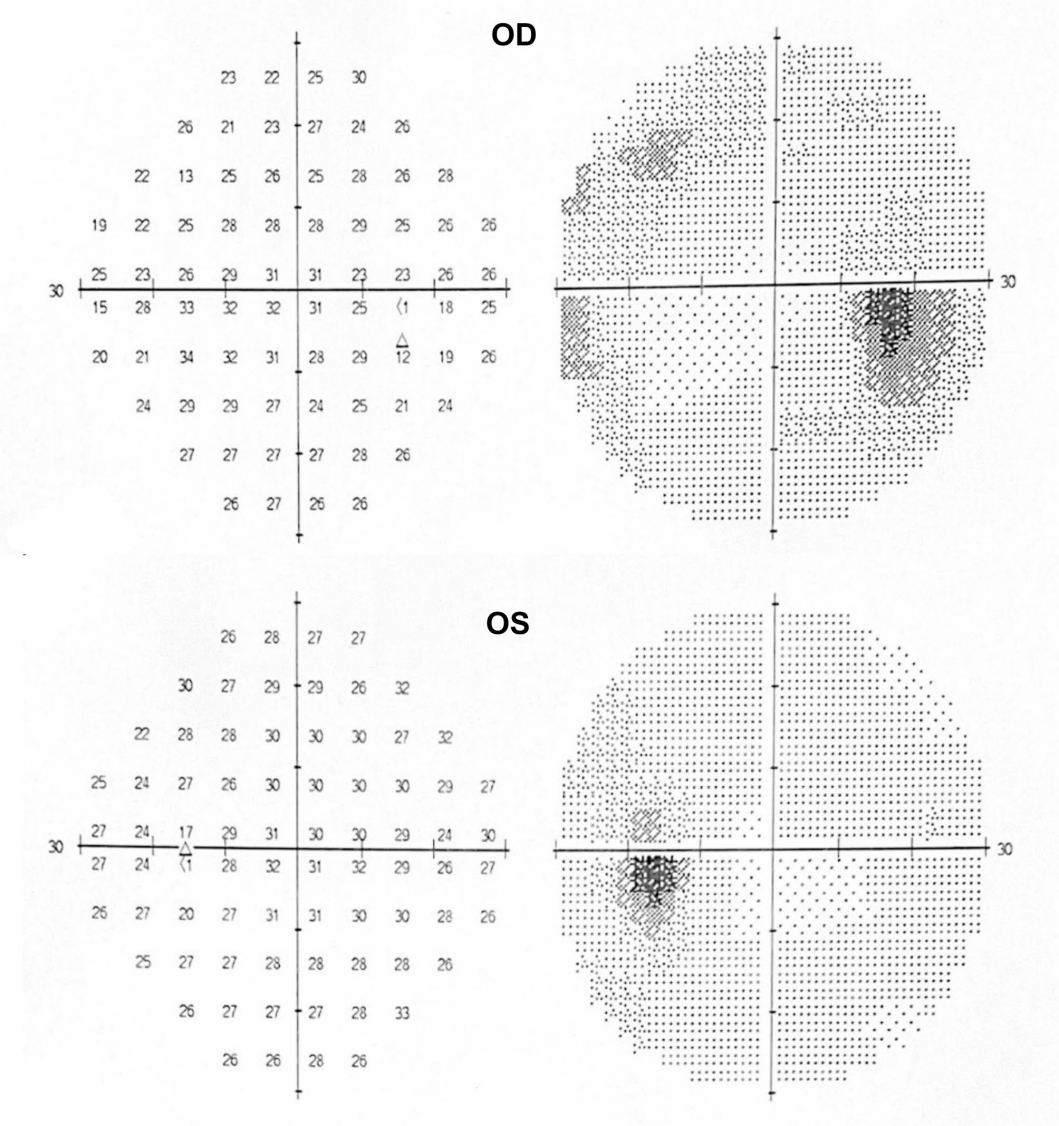
Bilateral visual fields: mild reduced sensitivity visual fields

We performed a comprehensive neuro-ophthalmic examination of the patient. Ocular examination revealed best-corrected visual acuity (BCVA) bilateral 20 of 20, with normal color vision and pupillary light responses. Extraocular motility testing showed no abnormality. Anterior segment examination was within normal limits. Fundus examination showed severe bilateral papilledema with blurred margins (Fig. 1). Bilateral Optical coherence tomography (OCT) images of optic discs showed intraretinal fluid tracking from the disc and abnormal retinal nerve fiber layer (RNFL) thickness increases. Automated visual field 30 − 2 revealed mild loss of sensitivity on the total deviation plot bilaterally (Fig. 2). The flash visual electrophysiology showed normal latency and amplitude in both eyes with 1.0 Hz.

Through careful inquiry of her medical history, the patient had a history of surgery for PT at another hospital one month before. According to the patient’s discharge medical report, the preoperative imaging examinations, neuro-ophthalmic examinations, and other regular examinations were normal. Under local anesthesia, the patient underwent a right skull base incision and right-side sigmoid sinus constriction surgery. The sigmoid sinus was pressed downward with a gelatin sponge. PT disappeared immediately after the surgery, but blurred vision, headache, and vomiting developed progressively.

**Fig. 3:**
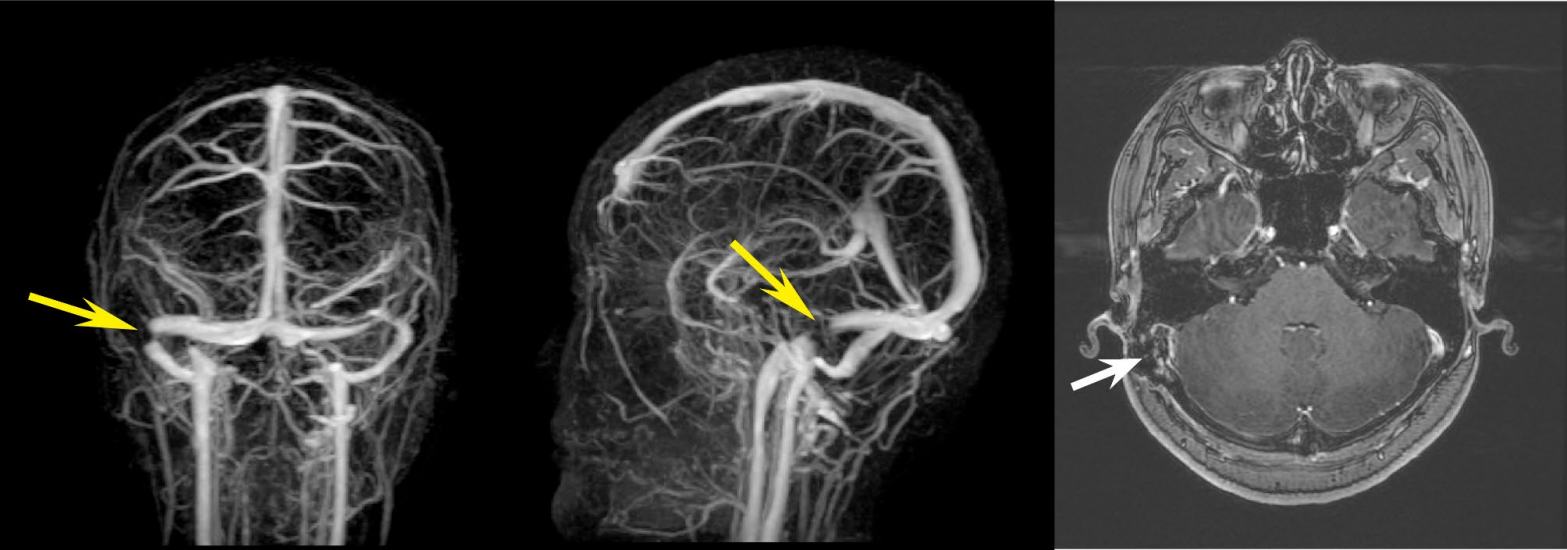
Magnetic resonance venography (MRV) showing the right sigmoid sinus filling defect shadow (yellow arrows), with a size of 13 mm*10mm. The same findings (white arrow) could be seen in contrast-enhanced magnetic resonance imaging (MRI)

Based on the history above, we repeated neuroimaging examinations on the patient (Fig. 3). Magnetic resonance venography (MRV) revealed the right sigmoid sinus postoperative stenosis. The patient underwent a lumbar puncture with an elevated cerebrospinal fluid (CSF) opening pressure of 29 cm H_2_O. CSF biochemical tests and cultures were negative. Laboratory studies were regular, including routine blood parameters, biochemical factors, conventional coagulation tests, infection indicators, C reactive protein, thyroid function, anti-nuclear antibody, and estradiol. The patient was diagnosed with intracranial hypertension and started on mannitol 50 g BID, furosemide 40 mg QD, and spironolactone 40 mg BID daily. However, she complained of progressive blurred vision with time.

**Fig. 4:**
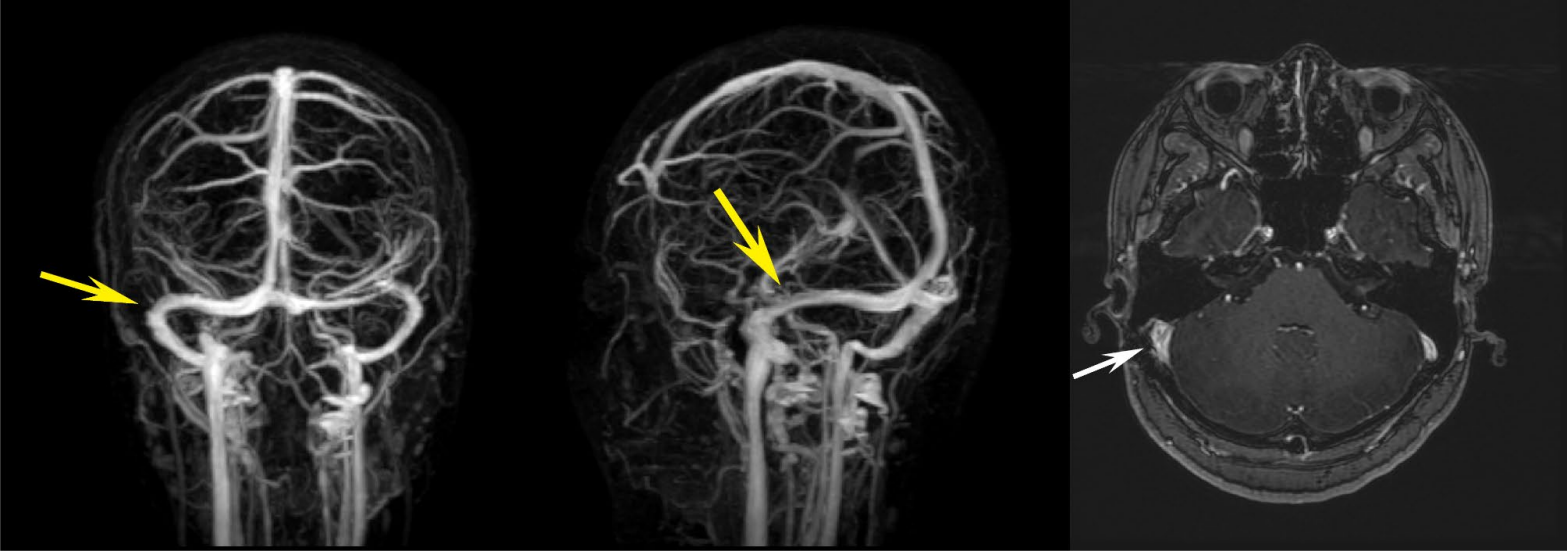
MRV (yellow arrows) and contrast-enhanced MRI (white arrow) showing resolved right sigmoid sinus stenosis after the second surgery

**Fig. 5:**
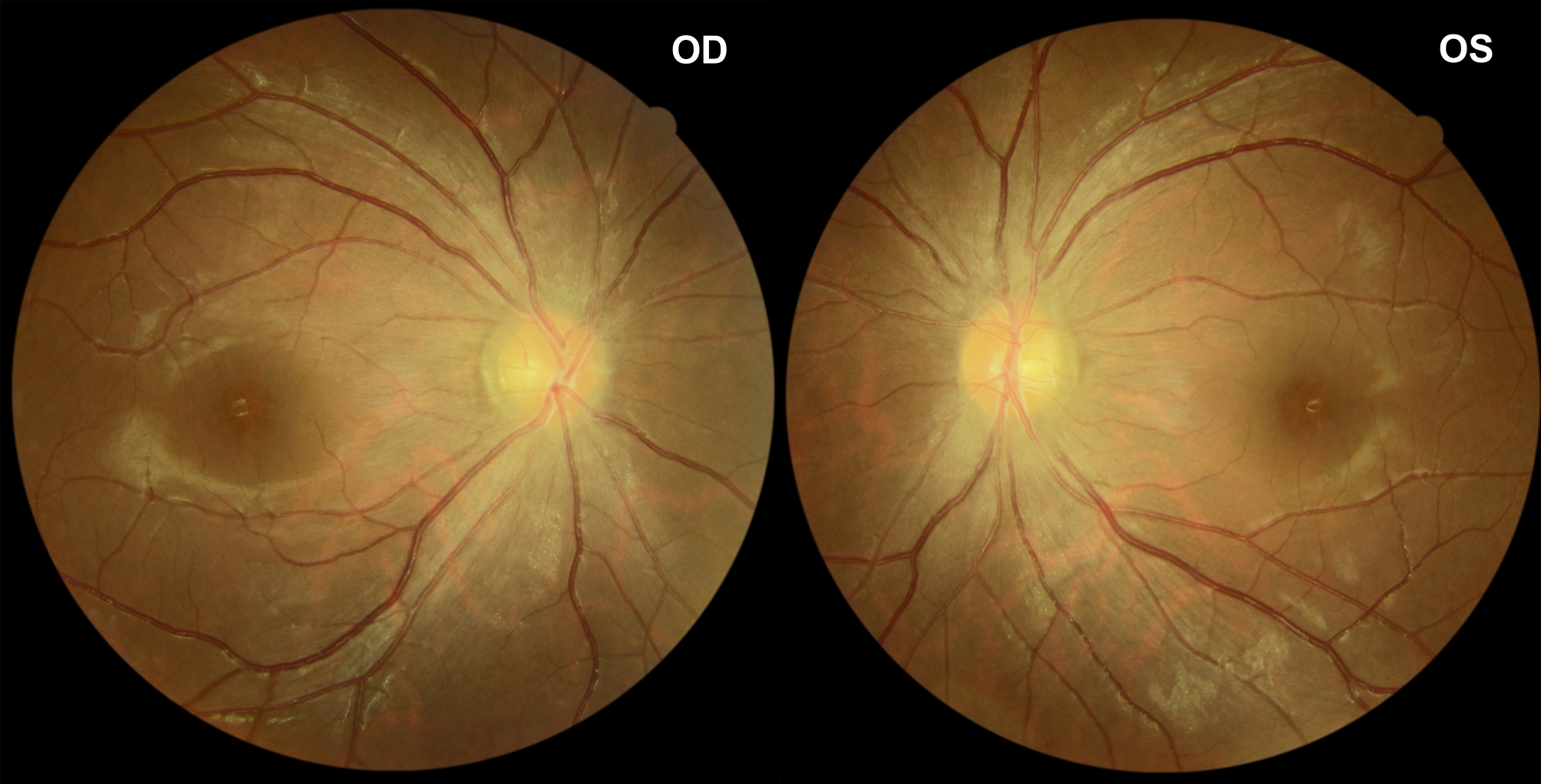
Follow-up ophthalmic investigations: funduscopic photographs of both eyes showing resolved papilledema

After discussing with the neurologists, we referred the patient to the initial surgeon, who repaired the right-side sigmoid sinus by removing the gelatin sponge implanted in the previous surgery. After the second surgery, her headache and vomiting disappeared immediately, while her blurred vision improved gradually. CSF opening pressure was 14.5 cm H_2_O at the 1-month follow-up. Ocular examination revealed BCVA 20 of 20 in both eyes. Bilateral papilledema was alleviated remarkably. Neuroimaging showed a well-visualized right-side sigmoid sinus (Fig. 4). There was no recurrence during the 3-year follow-up, and her bilateral papilledema resolved entirely (Fig. 5). Besides, there was also no recurrence of PT up to the present.

## Discussion and conclusions

Prolonged mechanical compression of optic nerve fibers caused by intracranial hypertension can result in irreversible vision damage and lead to blindness [1]. In general, patients with acute papilledema had better vision restored than patients with chronic, atrophic papilledema. The mechanism may be that the ICP lifting increases the pressure of the optic nerve tissues, inhibits the transport of axoplasmic flow, and finally results in the death of ganglion cells and axons [1]. The optic disc venous infarction secondary to intracranial hypertension may also play a role in visual function damage. Reducing ICP can save most cases of visual impairment before irreversible optic nerve ischemia occurs [3]. However, in certain cases, despite effective therapeutic management, axon loss might continue without any evidence of an atrophic optic nerve. Therefore, it is vital to identify the cause of intracranial hypertension, monitor and evaluate the changes in neuro-ophthalmic symptoms and signs, manage and control ICP, and avoid vision impairment as early as possible [1, 3].

For patients with continuous aggravation of symptoms and signs, neuroimaging and measurement of ICP should be performed as soon as possible to make an accurate diagnosis and appropriate management to save vision [1]. Treatment of intracranial hypertension is directed toward reducing ICP and preserving vision [7]. Carbonic anhydrase inhibitors (e.g., acetazolamide and topiramate) decrease ICP by reducing cerebrospinal fluid production [8]. In the acute setting, lumbar punctures and indomethacin administration are options. In patients with acute progressive vision loss, treatments with immediate effect, such as a CSF-diversion procedure, are considered the management [3]. Optic nerve sheath fenestration (ONSF) effectively improves severe papilledema and vision loss but is unhelpful for headaches as it cannot decrease ICP [9]. Venous stenting used to be an option for patients with venous sinus stenosis or obstruction who are not responding to medical therapy [3]. For patients with refractory IIH and concomitant venous sinus stenosis, venous stenting has become a safe and viable therapy option nowadays [10, 11]. In order to minimize complications of IIH related to venous stenting, surgeons are recommended to pay attention to the learning curve, hypercoagulable states, and obtaining internal carotid artery before and following stenting [10].

In our case, bilateral papilledema was caused by raised ICP related to right sigmoid sinus stenosis. Preoperative neuro-ophthalmic and neuroimaging examinations were normal. Symptoms and signs of intracranial hypertension appeared soon after the sigmoid sinus constriction surgery. Repeated MRV revealed a significant postoperative right sigmoid sinus stenosis (Fig. 3). These findings suggested that the patient presented with increased ICP associated with the surgery-induced right sigmoid sinus stenosis. Diuretic treatment could not relieve the symptoms and signs of intracranial hypertension. After discussing it with the neurologists, we considered solving postoperative sigmoid sinus stenosis the best choice [12]. With the development and popularization of neuroimaging, such as MRV and digital subtraction angiography (DSA), the venous sinus stenosis detection rate in patients with IIH is increasing [13, 14]. There is ongoing controversy about whether venous sinus stenosis is the cause or consequence of intracranial hypertension [4, 12, 15, 16]. Recent evidence suggests that restricted or collapsed dural sinuses frequently cause PT, which is frequently sustained by increased venous blood velocity-dependent flow turbulence [17]. Many of these instances harbor borderline opening pressures and lack papilledema. It is a pity whether the patient was IIH or SIH is unidentified due to the lack of lumbar puncture before the first surgery. Except for the right sigmoid sinus stenosis, the controlateral left sinus presented a smooth tapering narrowing, which reduces the sinus caliber to a thread-like flow (Fig. 3). The resulting overload of the dominant sinus was therefore crucial in this patient for the maintenance of an adequate venous drainage to prevent an excessive dural sinus pressure increase. There is evidence that bilateral dural sinus stenosis is a powerful risk factor for the development of raised ICP [18]. Rising dural sinus pressure leads to reduced CSF outflow rate via arachnoid villi and granulations with consequent CSF volume and pressure increase [19]. Nevertheless, there is no doubt that the sigmoid sinus constriction surgery must have impaired her cerebral venous drainage and worsened intracranial hypertension [4, 12, 20]. The marked improvement in symptoms and signs of intracranial hypertension after the second surgery confirmed our guess. Besides, the patient’s papilledema resolved entirely, and no recurrence was detected during the 3-year follow-up. Thus, the most likely cause of the onset of the cerebral hypertension condition with papilledema was dominant sinus surgical constriction by mechanical external compression, as confirmed by the complete clinical remission following the second operation to remove the implanted gelatin sponge. Therefore, we considered that the initial venous sinus constriction was an inappropriate therapeutic option for this patient with PT.

The most likely cause of PT for this patient was sinus stenosis. This causes blood acceleration in the stenosis with a turbulent motion during systole, resulting in the perception of a heartbeat. The first surgery’s clinical outcome supported this hypothesis. Because bilateral dural sinus stenosis would likely generate ICP, the first surgery was inappropriate and dangerous as there was stenosis on the left lateral sinus (Fig. 3). Indeed, this case highlights the relevance of adequate redundancy and stiffness of the dural sinus in CSF pressure homeostasis. It suggests that PT surgery by external sinus constriction is to be considered inappropriate. In fact, it implies the generation of a pressure gradient across the surgical stenosis, i.e., a condition currently considered the key factor for IIH pathogenesis. Even if surgical sinus constriction appears to alleviate PT, it achieves this by reducing flow turbulence, albeit at the expense of elevating sinus venous pressure further. Such pressure elevation is crucial in controlling ICP. Therefore, based on the current evidence available, this case report serves as an example of why surgical sinus constriction should no longer be considered a viable option for PT treatment.

To the best of our knowledge, this is the first report of increased ICP after sigmoid sinus constriction surgery. In summary, our case emphasized the importance of discovering the cause of papilledema and managing it appropriately in time. Despite the various and complicated causes of papilledema, thorough knowledge of the patient’s history and appropriate examination would be helpful in diagnosis. Ophthalmologists should strengthen cooperation with neurology, imaging, and other multidisciplinary departments to make early diagnoses and interventions to avoid severe vision impairment. Furthermore, this case also highlighted the importance of clarifying the etiology and selecting the appropriate therapeutic option for PT. Venous sinus constriction was an infeasible surgical option for PT.

## Data Availability

All data supporting our findings is contained within the manuscript.

## Abbreviations

BCVA: Best-corrected visual acuity
BMI: Body mass index
CSF: Cerebrospinal fluid
ICP: Increased intracranial pressure
IIH: Idiopathic intracranial hypertension
MRV: Magnetic resonance venography
OCT: Optical coherence tomography
ONSF: Optic nerve sheath fenestration
PT: Pulsatile tinnitus
RNFL: Retinal nerve fiber layer
SIH: Secondary intracranial hypertension
